# Interaction between ATM and PARP-1 in response to DNA damage and sensitization of ATM deficient cells through PARP inhibition

**DOI:** 10.1186/1471-2199-8-29

**Published:** 2007-04-25

**Authors:** Rocío Aguilar-Quesada, José Antonio Muñoz-Gámez, David Martín-Oliva, Andreína Peralta, Ma Teresa Valenzuela, Rubén Matínez-Romero, Rosa Quiles-Pérez, Josiane Menissier-de Murcia, Gilbert de Murcia, Mariano Ruiz de Almodóvar, F Javier Oliver

**Affiliations:** 1 Instituto López Neyra de Parasitología y Biomedicina López Neyra, CSIC. Granada, Spain; 2 IBIMER, Universidad de Granada, Granada, Spain; 3 Dept. Biología Celular, Universidad de Granada, Granada, Spain; 4 Hospital Universitario San Cecilio, Granada, Spain; 5 Département "Intégrité du Génome" de l'UMR 7175 École Supérieure de Biotechnologie de Strasbourg, Strasbourg, France

## Abstract

ATM and PARP-1 are two of the most important players in the cell's response to DNA damage. PARP-1 and ATM recognize and bound to both single and double strand DNA breaks in response to different triggers. Here we report that ATM and PARP-1 form a molecular complex *in vivo *in undamaged cells and this association increases after γ-irradiation. ATM is also modified by PARP-1 during DNA damage. We have also evaluated the impact of PARP-1 absence or inhibition on ATM-kinase activity and have found that while PARP-1 deficient cells display a defective ATM-kinase activity and reduced γ-H2AX foci formation in response to γ-irradiation, PARP inhibition on itself is able to activate ATM-kinase. PARP inhibition induced γ H2AX foci accumulation, in an ATM-dependent manner. Inhibition of PARP also induces DNA double strand breaks which were dependent on the presence of ATM. As consequence ATM deficient cells display an increased sensitivity to PARP inhibition. In summary our results show that while PARP-1 is needed in the response of ATM to gamma irradiation, the inhibition of PARP induces DNA double strand breaks (which are resolved in and ATM-dependent pathway) and activates ATM kinase.

## Background

The ATM protein kinase is centrally involved in the cellular response to ionizing radiation (IR) and other DNA double-strand-break-inducing insults. In persons affected with ataxia-telangiectasia (A-T), associated mutations in the ataxia-telangiectasia mutated (*atm*) gene render cells unable to cope with the genotoxic stresses from ionizing radiation and oxidative damage, thus resulting in a higher concentration of unrepaired DNA. Functional inactivation of the ATM gene product and Atm-null mice, which were created by disrupting the Atm locus, recapitulate the human A-T phenotype and display growth retardation, mild neurological dysfunction, male and female infertility, extreme predisposition to thymic lymphomas, and acute sensitivity to ionizing radiation [1-3].

ATM, the product of the ATM gene, is a member of a family of large proteins found in various organisms that share a COOH-terminal PI3 kinase-like domain. ATM has serine/threonine protein kinase activity and mediates the activation of multiple signal transduction pathways reviewed in [4-6].

Although it has been well established that IR exposure activates the ATM kinase domain, the actual mechanism by which ATM responds to damaged DNA has remained enigmatic until recently. Initial evidences indicated that ATM activation might involve autophosphorylation. A breakthrough in our understanding of this process came in a landmark publication by Bakkenist and Kastan [7]. They found that ATM molecules are inactive in undamaged cells, being held as dimers or higher-order multimers. In this configuration, the kinase domain of each molecule is blocked by the FAT domain of the other. Following DNA damage, each ATM molecule phosphorylates the other on a serine residue at position 1981 within the FAT domain, a phosphorylation that releases the two molecules from each other's grip, turning them into fully active monomers.

Poly(ADP-ribose) polymerase (PARP-1) is a nuclear enzyme which is activated in response to genotoxic insults by binding damaged DNA and attaching polymers of ADP-ribose to nuclear proteins at the expense of its substrate NAD+. The protein respond to DNA damage by transferring 50 to 200 molecules of ADP-ribose to various nuclear proteins, including transcription factors, histones and PARP-1 itself [8]. This poly(ADP-ribosyl)ation activity of PARP-1 appears to be important for maintaining genomic integrity [9] and it has been associated with longevity. Furthermore, PARP-1 is activated by agents infringing single stranded DNA damage such as alkylating agents, ionizing radiation, and oxidative damage.

A function of PARP-1 as a nick sensor has been proposed [10]. Its rapid activation upon DNA damage may result in poly (ADP-ribosyl)ation of key enzymes such as transducers of DNA damage, or alternatively, PARP-1 automodification could result in the recruitment of transducers to the damaged site. In this regard, a link between ATM and PARP-1 is supported by recent findings. One of these studies has demonstrated a sustained PARP-1 activation in ATM-/- cells due to the persistence of DNA damage [11,12]. Moreover, Menissier-de Murcia *et al*. [12], have reported that ATM and PARP-1 double deficient mice have a severe synergistic phenotypes leading to early embryonic lethality due to the effects of these proteins on signalling DNA damage and/or on distinct pathways of DNA repair. Furthermore, a recent study has shown that in vitro, PARP-1 inhibited the activation a subset of ATM substrates such as phosphorylation of p53 on serine 15 [13].

The aim of this study has been to elucidate the interaction between PARP-1 and ATM and how this partnership is involved in regulation of DNA repair pathways. We present evidences showing a physical association between ATM and PARP-1 in response to DNA damage as well as a poly (ADP-ribosyl)ation of ATM. The biological consequence of this interaction is a diminished activation of ATM-kinase in the absence of PARP-1. Surprisingly, preventing poly(ADP-ribosyl)ation with PARP inhibitors results in an increased constitutive ATM-kinase related to the PARP inhibitor's ability to induce DNA double strand breaks (DSBs) which were resolved in an ATM-dependent manner.

## Results

### PARP-1 interacts with ATM *in vivo *and ATM is modified by poly(ADP-ribosyl)ation

**Figure 1:**
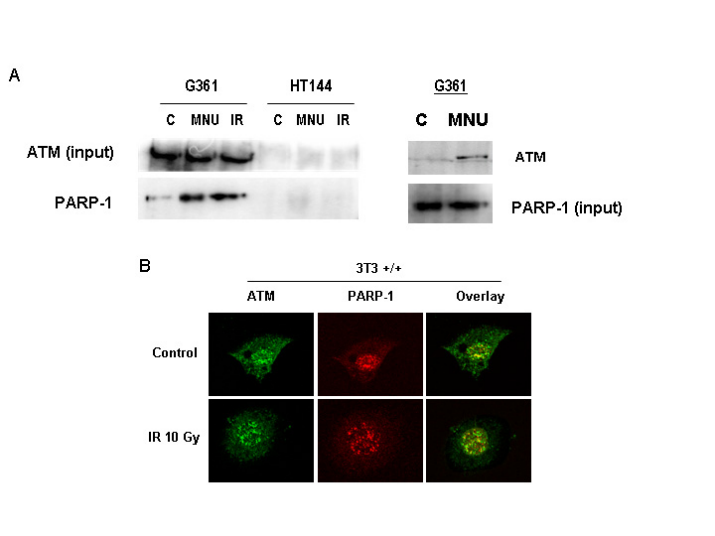
ATM form a complex with PARP-1 in DNA that is much more evident after treatment with DNA damaging agents. A: ATM was immunoprecipitated in human melanoma cell lines G361 and HT144 (ATM deficient) as explained under methods and the presence of PARP-1 was tested by immunoblot analysis. Cells were treated with 10 Gy IR or 2 mM MNU for 30 min. In the left panel, PARP-1 was immunoprecipitated from G361 cells treated or not with 2 mM MNU and western blot was performed to reveal ATM. B: Double indirect immunofluorescence in 3T3 fibroblasts (*parp-1*+/+)of PARP-1 (red signal) and ATM (green). Yellow signal correspond with co-localization of both proteins.

Previous studies have shown that PARP-1 and ATM double deficient mice are embryonic lethal very early during development, suggesting that the two proteins together are needed for the every day life of the animal [12]. In the present study our principal aim was to test whether these two proteins interact (both physical and functionally) in the response to γ-radiation. Figure 1A shows an ATM co-immunoprecipitation study using G361 cells (HT44, an ATM deficient cell line, was used as a negative control). ATM complexes were immunoprecipitated from nuclear extracts using an antibody against ATM (SYR 10G3/1) and the presence of PARP-1 was tested by immunoblot analysis using an anti-PARP-1 antibody. ATM form a tight complex with PARP-1 (1A, upper panels). Reciprocal immunoprecipitation experiments confirmed the previous observation (not shown). Interestingly, this complex was much more evident after DNA damage infringed with either the single alkylating agent N-methyl-N-nitrosourea (MNU, 2 mM) or 10 Gy of γ-irradiation. The interaction was direct and not mediated by DNA since the presence of ethidium bromide was not effective in abolishing the formation of the complex and the specificity of the pull-down was confirmed by the lack of co-immunoprecipitation using an IgG control (not shown). These results were confirmed by co-localisation studies with confocal microscopy, where after γ-irradiation the number of co-localised ATM/PARP-1 foci (yellow) increased respect to untreated cells. Both ATM and PARP-1 are localised in foci after DNA damage.

**Figure 2:**
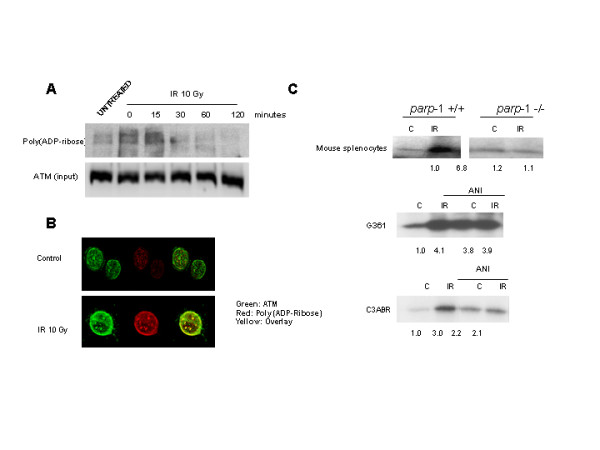
ATM is modified by poly(ADP)ribose. A: ATM was immunoprecipitated in G361 cells and the presence of poly(ADP ribose) was tested by immunoblot analysis in a time course, reaching a maximum after 15 min of IR (10 Gy). Equal loading was normalized by the input of ATM. B: Double indirect immunofluorescence in 3T3 fibroblasts (*parp-1*+/+) of poly(ADP-ribose) (red signal) and ATM (green). Yellow signal correspond with poly(ADP-ribosyl)ation of ATM. C: PARP-1 is needed for optimal activation of ATM. In vitro ATM-kinase assay. ATM activity increases after γ-irradiation in wild-type mouse splenocytes, but not in *parp*-1 knockout mouse splenocytes, where ATM is activated in control (upper panels). In G361 cells and C3ABR (middle and lower panel) irradiated with or without ANI co-treatment, ATM kinase activity increases after γ-irradiation and/or PARP inhibitors. Equal loading was checked with coomasie blue staining. Normalized signal respect to coomassie blue is shown below. Results are representative of three independent experiments.

In order to check whether ATM was modified or not by PARP-1 following DNA damage, the modification of ATM by PARP-1 was analysed in a time course experiment using the same antibody to immunoprecipitate ATM (figure 2A). ATM is, indeed, modified by poly (ADP-rybosil)ation and this modification increases during DNA damage, reaching a maximum after 30 min and then start to decrease. Polymer signal correspond to ATM molecular weight. Again, confocal microscopy confirmed the co-localisation of ATM and poly (ADP) ribose after ionizing radiation (figure 2B). In conclusion, these results are the first indication that ATM is physically associated to PARP-1 and is a substrate for this enzyme, co-localizing in the same foci after DNA damage.

### PARP-1 is needed for optimal activation of ATM

**Figure 3:**
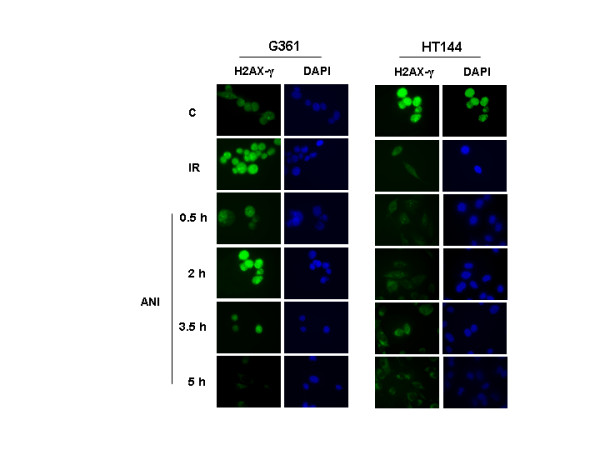
ATM is activated by PARP inhibitors. Immunostaining for γ-H2AX in G361 (ATM wild type) and HT144 (ATM deficient) cells exposed to γ-irradiation (positive control) or ANI (time course after drug exposure). H2AX (green signal) is phophorilated by ATM after ANI treatment in absence of γ-irradiation in G361 cells (ATM-proficient). DAPI is shown in blue and both views are in coincidence.

One key question derived from the previous results concern the functional consequences of the interaction between PARP-1 and ATM and between poly (ADP-ribosyl)ation and ATM on ATM activation. To address this question we have measured ATM kinase activity in wild type and PARP-1 deficient cells and in the presence and absence of the PARP inhibitor 4-amino,1–8,naphtalimide (ANI). Splenocytes from *parp-1*+/+ and *parp-1*-/- mice or G361 cells (a human melanoma cell line) were irradiated at 10 Gy. ATM was immunoprecipitated 30 minutes after the IR treatment and the ATM kinase assay performed (figure 2C). In these conditions ATM was strongly activated in response to ionizing radiation in *parp-1*+/+ splenocytes but not in *parp*-1-/- (Figure 3A). In G361 and C3ABR cells IR also activates ATM kinase but this increase was not observed if prior to IR there was a pre-incubation with the PARP inhibitor, ANI (figure 2C, middle and lower panel); surprisingly, the simple presence of the PARP inhibitor was able to activate ATM kinase.

### PARP inhibitors promote ATM activation through induction of DSBs

To gain insight about the increased basal ATM-kinase activity after the inhibition of PARP-1 with ANI (figure 2C), we performed an indirect inmunofluorescence against H2AX in its phosphorylated form, after the incubation with the PARP inhibitor in order to detect any DNA damage response. ATM wild type and deficient cells (G361 and HT144 respectively) were incubated with ANI at different times. Remarkably the sole fact of the incubation with ANI was able to elicits H2AX phosphorylation in wild type but not in ATM deficient cells. This effect was transient and reached a peak in 2 hours, declining afterwards (figure 3). γ-Irradiation was used as positive internal control (figure 3, upper panels). Previous results from our group have shown that PARP-1 null cells have a deficient p53 ser15 phosphorylation in response to ionising radiation [15], confirming with a different ATM substrate that ATM activation is compromised in the absence of PARP-1.

**Figure 4:**
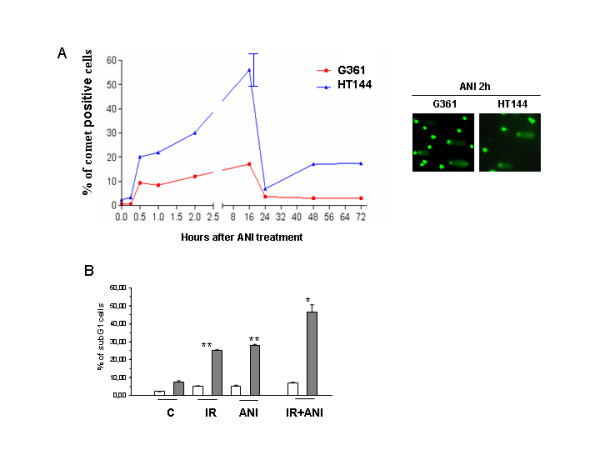
PARP inhibitors activate DSB repair by homologous recombination. A: neutral comet assay for detection of double strand breaks. For scoring the comet pattern, 800 nuclei from each slide were counted. HT144 ATM-deficient cells display more DSBs than G361 ATM-proficient cells after ANI treatment and only ATM wild type cells were able to completely resolve DSBs. Experiment is one representative of three similars. B: Analysis of cell death. Sub-G1 analysis was performed by flow cytometry using the propidium iodide (PI) DNA-staining method. Increased cell death with ANI is observed after long times of exposure (72 hours) in ATM-deficient cells (HT144, grey bars and ATM-proficient cells, G361, white bars) treated either with ANI alone or in combination with γ-irradiation respect ATM-proficient cells. Results represent the average ± SEM of three independent observations. * p < 0,05; **p < 0,01 respect to untreated HT144.

Recent results have shown that inhibition of PARP leads to stalled replication fork and the formation of DNA double strand breaks that are resolved by homologous recombination [17]. In order to test this possibility in our system we performed neutral comet assay to detect double strand breaks (DSB). DSB were produced by treatment with the PARP inhibitor in both ATM proficient (G361) and deficient (HT144) cells but only ATM wild type cells were able to completely resolve double strand breaks (figure 4A). PARP inhibition activates ATM through the induction of DSBs which are repaired by HR; since ATM deficient cells were less efficient in resolving DNA strand breaks that result from PARP inhibition we next examined their sensitivity to ANI in the presence and absence of IR. Results in figure 4B clearly show that ATM deficient cells are much more sensitive to PARP inhibition with or without further DNA damage.

## Discussion

Cells have evolved various sophisticated pathways to sense and overcome DNA damage as a mechanism to preserve the integrity of the genome. Environmental attacks like radiations or toxins, or spontaneous DNA lesions, trigger checkpoint activation and consequent cell cycle arrest leading to DNA repair or apoptosis. Two key proteins that coordinate recognition of DNA damage and signal transduction to p53 are ATM and PARP-1. ATM and PARP-1 participate in distinct forms of DNA repair that partially compensate for each other. PARP-1 and ATM participate in base excision repair (BER) and homologous recombination (HR), respectively. It is normally assumed that ATM signals for double strand breaks while PARP-1 participates in signalling from single DNA strand lesions. Here we report that these two proteins form a molecular complex that co-localizes in DNA damage foci.

Considerable evidence from *in vitro*, cell culture and *ex vivo *studies shows that poly(ADP-ribosyl)ation plays a critical role in the survival and maintenance of genomic stability of proliferating cells exposed to low or moderate levels of DNA-damaging agents [18]. The data presented in this study strongly support a role for PARP-1 and poly (ADP-ribose) in ATM activation: in the absence of PARP-1 there is a deficient ATM-kinase activation in response to ionizing radiation as measured by intrinsic kinase activity and H2AX phosphorylation. These results are in agreement with previous data showing that PARP-1 deficient mice are extremely sensitive to low doses of γ-radiation (as is the case for ATM-null mice), and this phenotype could be adscribed to a deficient ATM-kinase activation in tissues such as the intestine epithelium [9]. Also, results from our group have shown that p53 accumulation and p53-dependent gene activation are compromised in *parp*-1 knockout cells after γ-irradiation [15]. The insight of the consequences of the poly(ADP-ribosyl)ation of ATM are not clear yet since the inhibition of PARP induced indirectly DNA DSB, initiating new responses to DNA damage that interfere with elucidation of the activation of ATM.

PARP inhibitors have been used as radio and chemo-sensitizers in a number of experimental settings and a mechanism for DSB induction through the collision of unrepaired single DNA strand lesions with replication forks has been suggested [19,20]. Early reports claimed that the PARP inhibitor 3-aminobenzamide was a radio-sensitizer only in rodent cells [21] however more recently ANI (1000-fold more potent at inhibiting PARP activity compared with 3-aminobenzamide (3-ABA)) has been found to be radiation sensitizer to both rodent and human tumor cells [9,15,22]. The novel PARP inhibitor AG14361 has shown to increase the specificity and *in vivo *activity to enhance radiation therapy of human cancer through vasoactive effects and not directly in the cells in culture [23]. Therefore the question still remains open as to how human tumors could benefit from PARP inhibition during radiotherapy.

The second main conclusion in this study is that inhibition of PARP-1 activity leads to DSBs induction and activation of ATM and, at the same time, prevents IR-induced ATM-kinase activity. From the results presented here, there is a clear duality in the effect of PARP inhibition on ATM: while the lack of response to IR in ANI treated cells indicates that poly (ADP-rybosylation) of ATM is probably needed for optimal ATM activation, long term exposure to PARP inhibitor results in the generation of DSBs and secondarily in the activation of ATM kinase. DSBs generated in this way are due to stalled replication fork [24] and they are resolved by homologous recombination (HR), providing a therapeutic opportunity to specifically kill HR deficient tumor cells, as has been previously shown by different laboratories [25,26].

In summary, our study demonstrates a strong association between ATM and PARP-1 during the response to ionizing radiation, being PARP-1 and its activity needed for optimal activation of ATM kinase. On the other hand, inhibition of PARP leads to the activation of ATM kinase as result of the generation of DSBs, making ATM deficient cells particularly sensitive to PARP inhibitors.

## Conclusion

In this study we demonstrate the physical interaction between PARP-1 and ATM in response to ionizing radiation, the modification of ATM by poly(ADP-ribose) and the functional consequences of this interaction in PARP-1 deficient cells, where the activation of ATM kinase is compromised in response to IR. Additionally, PARP inhibition induces DNA double strand breaks who are resolved in an ATM-dependent manner. As result of that, ATM kinase is activated by PARP inhibition and ATM deficient cells are much more sensitive to PARP inhibition than ATM proficient cells.

## Methods

### Cell culture and treatments

We have used immortalised (3T3) murine embryonic fibroblasts expressing or lacking PARP-1 from *parp-1 *+/+ and -/- mice. G361 and HT144 are respectively wild type and A-T cell lines from a melanoma patient [14] (a kind gift from Dr. Y. Shiloh, Sackler School of Medicine, Tel Aviv).

Cells were in exponential growth at the time of IR treatment and the PARP inhibitor ANI was dissolved in culture medium at a concentration of 10 μM. It was added 60 min prior to IR. Irradiations, with or without ANI co-treatment, were performed using a ^60^Co source at a dose rate of 1.67 Gy/min. The used in all experiments was 10 Gy, unless otherwise stated.

### Indirect immunofluorescence

Immunostaining forATM, γ-H2AX, PARP-1 and PAR (poly(ADP-ribose)) were performed on cells plated onto coverslips and grown for 24 h before treatments. The medium was removed, the coverslips rinsed twice in PBS (37°C) and fixed in ice-cold methanol-acetone (1:1) for 10 minutes in the experiments with ATM, γ-H2AX, and in formaldehyde 4% for 10 minutes in the experiments with PARP-1 or PAR. The coverslips were rinsed twice in PBS-T (PBS containing 0,1% Tween-20) prior to incubation with primary antibody for 16 h at 4°C. The primary antibodies used in these experiments were: rabbit polyclonal IgG anti-phospho-H2AX (Ser139) (Upstate, Lake Placid, NY), mouse monoclonal IgG anti-PARP (Ab-2, Oncogene). anti-ATM mouse monoclonal antibody SYR 10G3/1 was a kind gift from Y. Shiloh (Tel Aviv University); anti-PAR rabbit polyclonal antibody was purchased from Biomol, Plymouth Meeting, PA. The coverslips were rinsed 3 times in PBS-T followed by a 45 min incubation at room temperature in the dark and then rinsed 4 × 5 min in PBS-T. The secondary antibodies used in this study were FITC-conjugated goat anti-rabbit IgG (Sigma, St. Louis, MO) and Cy3-conjugated sheep anti-mouse IgG (Sigma, St. Louis, MO) at a concentration of 1:400. Antibodies were diluted in PBS containing 1% bovine serum albumin and 5% goat serum. Nuclear counterstaining with 4',6'-diamidino-2-phenylindole dihydrochloride (DAPI) was performed after removal of excess secondary antibody. Slides were prepared using the Vectashield mounting medium (Vector Lab., Inc., Burlingame, CA 94010), coverslipped and stored in the dark at 4°C. Immunofluorescence images were obtained linear range of detection to avoid signal saturation using a Leica confocal microscopy.

### Immunoprecipitation and western blotting assay

Cell extracts, SDS-PAGE electrophoresis and western blotting were performed as previously described [15]. For immunoprecipitation, cell lysates were precleared by constant mixing for 2 hours with protein A-Sepharose (Pharmacia). The beads were removed by centrifugation, and the supernatant was mixed constantly overnight with a monoclonal antibody against ATM (SYR 10G3/1) or PARP-1 (Anti-PARP-1 Ab-2, Oncogene). Immune complex were adsorbed onto protein A-Sepharose, boiled and electrophoresed on polyacrylamide gels. The membranes were probed with antibodies directed against ATM (SYR 10G3/1, Tel Aviv University), PARP-1 (Anti-PARP-1 Ab-2, Oncogene) and Poli (ADP-Ribose) (Biomol).

### In Vitro ATM-kinase assay

ATM kinase assays were conducted using the protocol described by Sarkaria *et al*. [16]. ATM was immunoprecipitated from G361 cell extracts and from *parp-1 +/+ *and *parp-1 -/- *murine spleen extracts. Briefly cell extracts were prepared by resuspending cells in lysis buffer (20 mM HEPES pH = 7.4, 150 mM NaCl, 1.5 mM MgCl_2_, 1 mM EGTA, with protease inhibitors and 0.2% Tween during 20 minutes at 4°C. The lysates were clarified by centrifugation and immunoprecipitations were carried out with SYR 10G3/1 anti-ATM mouse monoclonal antibody. Then immune complex were adsorbed onto protein A-Sepharose and washed twice with kinase buffer (10 mM HEPES pH = 7.4, 50 mM NaCl, 10 mM MgCl_2_) and once with a high salt buffer (0.1 M Tris-HCl pH = 7.4, 0.6 M NaCl). Kinase reactions were initiated with the addition of an equal volume of kinase buffer containing PHAS-I (20 ng/ml), 10 mM MnCl_2_, 1 mM DTT and 10 mM [^32^P]ATP. Kinase reactions were performed at 30°C during 20 minutes and terminated by the addition of 6×SDS loading buffer (1:1), and reaction products were resolved by SDS-PAGE. Incorporation of ^32^P into the PHAS-I substrate was evaluated by phosphorimaging. All kinase reactions were performed under linear reaction conditions. Equal loading in each lane was guaranteed by coomasie blue staining.

## Abbreviations

ATM, ataxia telanagiectasia

BER, base excision repair

DAPI, 4',6'-diamidino-2-phenylindole dihydrochloride

DSB, double strand break

DTT, 1,4-dithiothreitol

EGTA, ethylene glycol tetraacetic acid

HEPES, 4-(2-hydroxyethyl)-1-piperazineethanesulfonic acid)

HR, homologous recombination

MNU, N-methyl-N-nitrosourea

PAR, poly(ADP-ribose)

PARP, poly(ADP-ribose) polymerase

SDS-PAGE, sodium dodecyl sulphate-polyacrylamide gel electrophoresis

## Authors' contributions

RA-Q carried out poly(ADP-ribosylation) studies, immunofluorescence, ATM kinase, comet assay and apoptosis studies; JAMG contributed to apoptosis studies; DMO helped with ATM kinase assay; AP, MTV, RMR and RQP contributed to immunoprecipitation and comet assays studies. JMM, GdM, MRdA conceived and participated in design the study. FJO conceived of the study, and participated in its design and coordination. All authors read and approved the final manuscript.
